# Cardio-ankle vascular index is associated with cardiovascular target organ damage and vascular structure and function in patients with diabetes or metabolic syndrome, LOD-DIABETES study: a case series report

**DOI:** 10.1186/s12933-014-0167-y

**Published:** 2015-01-16

**Authors:** Manuel Ángel Gómez-Marcos, José Ignacio Recio-Rodríguez, María Carmen Patino-Alonso, Cristina Agudo-Conde, Leticia Gómez-Sánchez, Marta Gomez-Sanchez, Emiliano Rodríguez-Sanchez, Jose Angel Maderuelo-Fernandez, Luís García-Ortiz, LOD-DIABETES Group

**Affiliations:** Primary Care Research Unit, the Alamedilla Health Center, Avda. Comuneros 27, 37003 Salamanca, Spain; Medicine Department, University of Salamanca, Salamanca, Spain; Statistics Department, University of Salamanca, Salamanca, Spain

**Keywords:** Target organ damage, Cardio ankle vascular index, Vascular structure, Vascular function, Cardiovascular risk, Diabetes mellitus type 2, Metabolic syndrome

## Abstract

**Background:**

The cardio ankle vascular index (CAVI) is a new index of the overall stiffness of the artery from the origin of the aorta to the ankle. This index can estimate the risk of atherosclerosis. We aimed to find the relationship between CAVI and target organ damage (TOD), vascular structure and function, and cardiovascular risk factors in Caucasian patients with type 2 diabetes mellitus or metabolic syndrome.

**Methods:**

We included 110 subjects from the LOD-Diabetes study, whose mean age was 61 ± 11 years, and 37.3% were women. Measurements of CAVI, brachial ankle pulse wave velocity (ba-PWV), and ankle brachial index (ABI) were taken using the VaSera device. Cardiovascular risk factors, renal function by creatinine, glomerular filtration rate, and albumin creatinine index were also obtained, as well as cardiac TOD with ECG and vascular TOD and carotid intima media thickness (IMT), carotid femoral PWV (cf-PWV), and the central and peripheral augmentation index (CAIx and PAIx). The Framingham-D’Agostino scale was used to measure cardiovascular risk.

**Results:**

Mean CAVI was 8.7 ± 1.3. More than half (54%) of the participants showed one or more TOD (10% cardiac, 13% renal; 48% vascular), and 13% had ba-PWV ≥ 17.5 m/s. Patients with any TOD had the highest CAVI values: 1.15 (CI 95% 0.70 to 1.61, p < 0.001) and 1.14 (CI 95% 0.68 to 1.60, p < 0.001) when vascular TOD was presented, and 1.30 (CI 95% 0.51 to 2.10, p = 0.002) for the cardiac TOD. The CAVI values had a positive correlation with HbA1c and systolic and diastolic blood pressure, and a negative correlation with waist circumference and body mass index. The positive correlations of CAVI with IMT (β = 0.29; p < 0.01), cf-PWV (β = 0.83; p < 0.01), ba-PWV (β = 2.12; p < 0.01), CAIx (β = 3.42; p < 0.01), and PAIx (β = 5.05; p = 0.04) remained after adjustment for cardiovascular risk, body mass index, and antihypertensive, lipid-lowering, and antidiabetic drugs.

**Conclusions:**

The results of this study suggest that the CAVI is positively associated with IMT, cf-PWV, ba-PWV, CAIx, and PAIx, regardless of cardiovascular risk and the drug treatment used. Patients with cardiovascular TOD have higher values of CAVI.

**Trial registration:**

Clinical Trials.gov Identifier: NCT01065155

## Background

The early detection of atherosclerosis is important for patients with type 2 diabetes mellitus (T2DM) or metabolic syndrome [1], because cardiovascular disease is a main cause of death in these people [2,3].These pathologies are associated with more cardiovascular risk factors [4,5], more comorbidities [6,7], and more renal [8], cardiac [9,10], and vascular [11] target organ damage (TOD). They are also associated with premature vascular aging and altered parameters assessing vascular structure, such as the ankle brachial index (ABI) [12] or carotid intima media thickness (IMT) [11]. They also occur with increased arterial stiffness [13] based on carotid femoral pulse wave velocity (cf-PWV), or with high brachial-ankle pulse wave velocity (ba-PWV) could predict all-cause mortality and cardiovascular events in subjects with diabetes [14,15], increased ba-PWV was significantly correlated with cardiac autonomic neuropathy and with subclinical myocardial injury in patients with type 2 diabetes [16].They are also associated with pulse wave parameters such as the central and peripheral augmentation indices (CAIX and PAIx) [17-19].

The cardio-ankle vascular index (CAVI) is a new index [20,21] of the overall stiffness of the artery from the origin of the aorta to the ankle, and it is able to estimate the risk of atherosclerosis [22]. The arterial stiffness estimated using CAVI in healthy subjects increases linearly with aging, and is higher in men than in women [23]. In patients with risk factors such as diabetes or obesity [24-26], the CAVI values are higher than in controls. CAVI is associated with carotid and coronary atherosclerosis [27-29]. The CAVI method is a useful tool to screen people with moderate to advanced levels of atherosclerosis [30].

Even though there is much evidence supporting the relationship between CAVI and cardiovascular risk factors and atherosclerosis, most studies have been done on Asian populations. The clinical relevance of this work is given because it is the first study to analyze in the same sample, the association between CAVI and cardiovascular risk factors, TOD (renal, vascular, and heart), other parameters of vascular structure and function, and the thickness of the retinal vessels in Caucasian patients with increased insulin resistance. Therefore, the aim of this study is to analyze the association of CAVI with target organ damage, vascular structure and function, and cardiovascular risk factors in Caucasian patients with T2DM or metabolic syndrome.

## Methods

### Study design

This study analyzed 110 subjects in the 4th year of follow up who were included in the longitudinal LOD-DIABETES study (NCT01065155) [31].

### Study population

Using consecutive sampling, we included 112 patients who visited their family doctor from January 2009 to January 2010 with T2DM (n = 68), which was defined using the American Diabetes Association criteria [32] or metabolic syndrome (n = 44) (defined according to the National Cholesterol Education Program, ATP III1 definition [33]). The subjects were sampled from a population of 46000 people from two primary care centers (including 2412 diagnosed with diabetes and 4100 with metabolic syndrome). The exclusion criteria were patients unable to comply with the protocol requirements (psychological and/or cognitive disorders, failure to cooperate, educational limitations, problems with understanding written language, failure to sign the informed consent document), patients participating or planning to participate in a clinical trial during the study, and patients with serious comorbidities representing a threat to life over the subsequent 12 months. Patients with a history of cardiovascular disease were not excluded from the study. A sample-size calculation indicated that the 110 patients included in the study constituted a sufficient sample for detecting a correlation coefficient of 0.26 between CAVI and IMT in a two-sided test, with a significance level of 95% and power of 80% (EPIDAT 4.0). The study was approved by an independent ethics committee of Salamanca University Hospital (Spain), and all participants gave written informed consent according to the general recommendations of the Declaration of Helsinki [34].

### Measurements

A detailed description has been published elsewhere regarding how the clinical data were collected, the anthropometric measurements were made, blood pressure was recorded, TOD was assessed, and the analytical parameters were obtained [31].

### Office blood pressure

Office blood pressure (BP) was calculated as the average of the last two of three measurements of systolic blood pressure (SBP) and diastolic blood pressure (DBP) made with a validated sphygmomanometer (OMRON Model M10-IT). Measurements were made on the dominant arm of participants in the seated position after at least 5 minutes of rest, with a cuff of appropriate size as determined by measurement of the upper-arm circumference and following the recommendations of the European Society of Hypertension [35].

### Vascular assessment

Cardio ankle vascular index (CAVI), brachial ankle pulse wave velocity (ba-PWV), and ankle/brachial index (ABI) were measured using a VaSera VS-1500® device (Fukuda Denshi). The ba-PWV was calculated, as was CAVI, which gives a more accurate calculation of the degree of atherosclerosis. CAVI integrates the cardiovascular elasticity derived from the aorta to the ankle pulse velocity through an oscillometric method, it is used as a good measure of vascular stiffness, and it does not depend on BP [21]. CAVI values were automatically calculated by substituting the stiffness parameter β in the following equation to detect the vascular elasticity and the brachial ankle PWV: Stiffness parameter β = 2ρ × 1/ (Ps –Pd) × ln (Ps/Pd) × ba-PWV^2^, where ρ is the blood density, Ps and Pd are SBP and DBP in mmHg, respectively, and the ba-PWV is measured between the aortic valve and the ankle. The average coefficient of the variation of the CAVI is less than 5%, which is small enough for clinical use and confirms that CAVI has favorable reproducibility [20,23]. CAVI was measured at rest and considered normal (CAVI < 8), borderline (8 ≤ CAVI < 9), or abnormal with subclinical atherosclerosis (CAVI ≥9). ba-PWV ≥ 17.5 was considered abnormal [36,37]. The higher obtained CAVIs and ba-PWV were considered for the study.

### Carotid femoral pulse wave velocity (cf-PWV) and peripheral (PAIx) and central augmentation index (CAIx)

These parameters were estimated using the SphygmoCor System (AtCor Medica lPty Ltd., Head Office, West Ryde, Australia). The central augmentation index (CAIx) is a composite index that integrates the amount of the wave that is reflected back to the aorta depending on the tone of the resistance arteries, which are the main peripheral reflecting sites. This system (Px Pulse Wave Analysis) was used with the patient in the sitting position and resting the arm on a rigid surface. Pulse wave analysis was performed with a sensor in the radial artery using mathematical transformation to estimate the aortic pulse wave. The reliability of these measurements was evaluated before the study using the CAIx intra-class correlation coefficient (ICC), which showed values of 0.97 (95% CI: 0.94-0.99) for intra-observer agreement in repeated measurements of 22 subjects. According to the Bland-Altman analysis, the mean difference for intraobserver agreement (95% limits of agreement) was 0.45 (−9.88-10.79). From the morphology of the aortic wave, CAIx was estimated using the following formula: increase in central pressure × 100/pulse pressure. The value was adjusted to a heart rate of 75 by the SphygmoCor System device.

The peripheral augmentation index (PAIx) is a measurement taken directly from the late systolic shoulder of the peripheral arterial waveform. The index is defined as the ratio of the difference in amplitude between the second peak and diastolic pressure to the difference between the first peak and diastolic pressure [18]. The PAIx was calculated to yield a percent (%) value as follows: (second peak systolic blood pressure [SBP2] - diastolic blood pressure [DBP])/(first peak SBP - DBP) × 100 [18]. The pulse waves of the carotid and femoral arteries were analyzed using the SphygmoCor System with the patient in a supine position. The delay was estimated with respect to the ECG wave and calculating PWV. Distance measurements were taken with a measuring tape from the sternal notch to the carotid and femoral arteries at the sensor location [35]. Subclinical organ damage was defined as cf-PWV >12 m/s [35].

### Assessment of vascular structure by carotid intima media thickness (IMT)

Carotid ultrasound to assess carotid IMT was performed by two investigators trained for this purpose before starting the study. The reliability of the recordings was evaluated before the study using the intra-class correlation coefficient, which showed values of 0.97 (95% CI: 0.94 to 0.99) for intra-observer agreement in repeated measurements on 20 subjects, and 0.90 (95% CI: 0.74 to 0.96) for inter-observer agreement. According to the Bland-Altman analysis, the mean difference for interobserver agreement (95% limits of agreement) was 0.01 (−0.03 to 0.06). A Sonosite Micromax ultrasound device paired with a 5–10 MHz multi-frequency high-resolution linear transducer with Sonocal software was used for performing automatic measurements of IMT in order to optimize reproducibility.

Measurements were made of the common carotid after the examination of a 10-mm longitudinal section at a distance of 1 cm from the bifurcation. They were performed in the anterior or proximal wall and in the posterior or distal wall in the lateral, anterior, and posterior projections. The measurements were taken following an axis perpendicular to the artery to discriminate two lines: one for the intima-blood interface and the other for the media-adventitious interface. A total of 6 measurements were obtained of the right carotid, with another 6 measurements of the left carotid. Average values (average IMT) automatically calculated by the software were used [38]. The measurements were obtained with the subject lying down, with the head extended and slightly turned opposite to the examined carotid artery. Average IMT was considered abnormal if > 0.90 mm, if there were atherosclerotic plaques with a diameter of 1.5 mm, or if there was a focal increase of 0.5 mm or 50% of the adjacent IMT [35].

### Evaluation of retinal vessels

Retinography was performed using a Topcon TRC NW 200 non-mydriatic retinal camera (Topcon Europe B.C., Capelle a/d Ijssel, The Netherlands). Nasal and temporal images centered on the disk were obtained. The nasal image with the centered disk was loaded into developed software called the arteriolar-venular (AV) diameters index calculator (Ciclorisk SL, Salamanca, Spain, registry no. 00/2011/589). The software automatically recognizes the disk and draws two external concentric circles which delimit area A, which is between 0 and 0.5 disk diameters from the optic disk margin, and area B, which is between 0.5 and 1 disk diameters from the margin.

The software first identifies the limits of the different vessels and then automatically recognizes arteries and veins. Then, it makes multiple measurements of the diameter of the section of the vessels circulating through area B. It finally estimates the mean caliber of veins and arteries in mm, and these measurements are summarized as an arteriole-venule ratio (AVR). An AVR of 1.0 suggests that arteriolar diameters are the same on average as venular diameters in the eye, whereas a smaller AVR suggests narrower arterioles [39].

We used pairs of the main vessels in the upper and lower temporal quadrants, rejecting all other vessels, to improve reliability and increase efficiency of the process. Measures are analyzed for each quadrant separately and together to estimate the mean measure in each eye. The reliability of such recordings was evaluated before the study using the ICC, which showed values of 0.998 (95% CI: 0.997 to 0.999) for vein caliber, 0.969 (95% CI 0.940–0.984) for arteries, and 0.981 (95% CI 0.965–0.990) for AVR intraobserver ICC, which was measured using a subsample of 40 photographs. The estimated average time to analyze a patient (two photographs) was less than 2 min [40].

### Renal assessment

Kidney damage was assessed by measuring plasma creatinine concentration. Glomerular filtration rate (GFR) was estimated according to the Modification of Diet in Renal Disease-Isotopic Dilution Mass Spectrometry (MDRD-IDMS) [41], and proteinuria was assessed from the albumin/creatinine ratio. TOD was defined according to the criteria in the 2007 European Society of Hypertension/European Society of Cardiology Guidelines [42].

### Cardiac assessment

The electrocardiographic examination was performed using a General Electric MAC 3.500 ECG System (General Electric, Niskayuna, NY, USA), which automatically measures the voltage and duration of waves and estimates the criteria of the Sokolow and Cornell voltage-duration product (Cornell VDP). TOD was defined according to criteria in the 2007 European Society of Hypertension/European Society of Cardiology Guidelines [42].

### Cardiovascular risk assessment

Risk of cardiovascular morbidity and mortality was estimated using the published Framingham-D’Agostino risk equation [43]. Risk factors for morbidity and mortality used by the Framingham-D’Agostino scale include age, total cholesterol, high-density lipoprotein cholesterol, and SBP as quantitative variables. Sex, drug treatment for hypertension, smoking, and history of diabetes mellitus are dichotomous variables. We considered patients to be at high risk when the scale was ≥20% for the next 10 years. The individuals performing the different tests were blinded to the clinical data of the patients. All assessments were made within a period of 10 days.

### Statistical analysis

Continuous variables were expressed as the mean ± standard deviation for normally distributed continuous data, the median (interquartile range, IQR) for asymmetrically distributed continuous data, and the frequency distribution for categorical data. Statistical normality was tested using the Kolmogorov–Smirnov test. Quantitative variables were compared using the Student t-test or Mann–Whitney U-test as appropriate. A partial correlation was examined between CAVI with cardiovascular risk factors and target organ damage, controlling for age, gender, and antihypertensive, lipid-lowering, and antidiabetic drugs. We performed multiple linear regression analyses with CAVI as the independent variable and IMT mean, CAIx, PAIx, AVR, cf-PWV, and ba-PWV as dependent variables. We adjusted by Framingham-D’Agostino cardiovascular risk, body mass index (BMI), and antihypertensive, lipid-lowering, and antidiabetic drugs. The data were analyzed using the Statistical Package for the Social Sciences version 20.0 (SPSS, Chicago, IL, USA). A value of p < 0.05 was considered statistically significant.

## Results

Throughout the fourth year of study of follow-up, two males died as a result of acute myocardial infarction: one with T2DM and the other with metabolic syndrome (aged 76 and 65 years, respectively).

Table 1 shows the demographics and clinical characteristics, cardiovascular risk factors, and cardiovascular risk estimated with the Framingham-D’Agostino scale. The mean age was 61.2 ± 11.1 years (women 50.6; men 62.2), and 37.3% of the 110 subjects were women.

**Table 1:** **Baseline demographic and clinical characteristics of patients**

**Variable**	**Mean/Median/Number n**	**SD/IQR/ (%)**
Age (years)	61.2	11.1
Female sex n (%)	41	37.3
Smoking n (%)	19	17.6
Ischemic heart disease n (%)	11	10
Cerebrovascular disease n (%)	3	3.9
Waist circumference (cm)	102.9	11.9
Body mass index (kg/m^2^)	30.2	4.8
Obesity n (%)	55	50.9
Office systolic blood pressure (mmHg)	132	16
Office diastolic blood pressure (mmHg)	77	10
Office pulse pressure (mmHg)	56	15
Heart rate (beats/min)	68	11
Hypertension n (%)	90	81.8
Antihypertensive drugs n (%)	88	81.5
Serum glucose (mg/dL)	103	89.3-128.3
HbA1c (%)	6.3	5.7-7.0
Diabetes n (%)	71	64.5
Antidiabetic drugs n (%)	65	60.2
Total cholesterol (mg/dL)	185.3	31.9
Triglycerides (mg/dL)	128	95.0-162.5
High density lipoprotein cholesterol (mg/dL)	50.2	11.8
Low density lipoprotein cholesterol (mg/dL)	109.5	29.7
Dyslipidemia n (%)	68	63.6
Metabolic syndrome n (%)	39	35.5
Lipid lowering drugs n (%)	70	64.8
Cardiovascular risk Framingham D’Agostino	25.3	19.5

Values are means and standard deviations (SD) for normally distributed continuous data, medians and interquartile range (IQR) for asymmetrically distributed continuous data and absolute frequency and proportions for categorical data.

Table 2 shows the TOD and the parameters used to assess the vascular structure and function. Among the patients, 54% had one or more TOD: 10% cardiac, 13% renal, and 48% vascular. 13% had ba-PWV ≥ 17.5 m/s. The CAVI value was 8.71 ± 1.28 in men and 8.65 ± 1.40 in women.

**Table 2:** **Values of organ damage markers and vascular structure and function parameters**

**Variable**	**Mean/Median/Number n**	**SD/IQR/ (%)**
Serum creatinine (mg/dL)	0.9	0.7-1
Target organ damage creatinine n (%)	4	3.7
GFR with MDRD-IDMS (mL/min/1.73 m^2^)	89.9	19.8
Target organ damage (GFR <60) n (%)	6	5.6
Albumin/creatinine (mg/g)	2.58	0.0-8.88
Target organ damage (Albumin/creatinine) n (%)	10	9.3
Target organ damage renal n (%)	14	13.1
Cornell VDP (mmms)	1604.1	645.3
Sokolow (mm)	20.1	6.4
Target organ damage heart n (%)	11	10.3
Ankle/brachial index	1.14	0.11
Target organ damage ankle/brachial index n (%)	5	4.7
Carotid Intima-media thicknes average mean (mm)	0.78	0.12
Target organ damage Carotid n (%)	36	33.6
cf-PWV (m/sec)	9.6	2.6
Target organ damage Pulse Wave Velocity n (%)	30	28.0
Target organ damage Vascular n (%)	51	47.7
Target organ damage global	58	54.2
Arteriovenous índex	0.81	0.13
Arteriolar caliber mean (μm)	106.66	13.17
Venular caliber mean (μm)	138.55	16.80
Central Augmentation Index	27.09	13.21
Peripheral Augmentation index	90	78-102.5
ba-PWV (m/sec)	14.76	3.09
ba-PWV ≥17.5 (m/sec)	14	13.3
Cardio-Ankle Vascular Index.	8.70	1.31

Values are means and standard deviations (SD) for normally distributed continuous data, medians and interquartile range (IQR) for asymmetrically distributed continuous data and absolute frequency and proportions for categorical data.

GFR: Glomerular filtration rate. MDRD-IDMS: Modification of Diet in Renal Disease-Isotopic Dilution Mass Spectrometry. VDP: Voltage–Duration Product. cf-PWV: carotid femoral Pulse Wave Velocity. ba-PWV: brachial ankle Pulse Wave Velocity.

Subjects with CAVI ≥ 9 were older and had higher values of HbA1c, office systolic blood pressure, cardiovascular risk, IMT, cf-PWV, ba-PWV, and PAIx, and they had lower values of total and LDL cholesterol (Table 3).

**Table 3:** **Values of cardiovascular risk factors, organ damage markers and vascular structure and function parameters according to CAVI value**

**Value of CAVI**	**CAVI < 9 (58%) Mean/Median/SD/IQR**	**CAVI ≥ 9 (42%) Mean/Median/SD/IQR**	**p-value**
Age (years)	56.5 ± 11.4	67.7 ± 7.3	<0.01
Waist circumference (cm)	102.9 ± 10.7	102.2 ± 12.6	0.77
Body mass index (kg/m2)	30.6 ± 4.2	29.2 ± 5.1	0.13
Total Cholesterol (mg/dL)	191.7 ± 28.1	176.6 ± 35.0	0.02
LDL cholesterol (mg/dL)	115.0 ± 28.3	102.2 ± 30.7	0.03
Tryglicerides (mg/dL)	128 (96.0-161.5)	113.5 (82.5-159.5)	0.57
HDL cholesterol (mg/dL)	50.6 ± 12.0	50.1 ± 11.5	0.85
Lipid lowering drugs n (%)	32 (52.5)	35 (79.5)	<0.01
Diabetics n (%)	32 (52.5)	35 (79.5)	<0.01
Serum glucose (mg/dL)	96 (85.5-120.5)	107 (93.0-130.8)	0.05
HbA1c	6.1 (5.6-6.5)	6.6 (5.9-7.1)	0.03
Antidiabetic drugs n (%)	31 (50.8)	31 (70.5)	0.05
Office SBP (mm Hg)	127 ± 14	139 ± 17	<0.01
Office DBP (mm Hg)	77 ± 9	78 ± 10	0.59
Antihypertensive Drugs n (%)	50 (82.0)	36 (81.8)	0.98
CVR Framingham D’Agostino	20.5 ± 17.1	32.0 ± 20.8	<0.01
Serum creatinine (mg/dL)	0.9 (0.8-1.0)	0.8 (0.7-1.0)	0.69
GFR with MDRD-IDMS (mL/min/1.73 m2)	92.3 ± 19.1	87.6 ± 20.0	0.22
Albumin/creatinine (mg/g)	2.97 (0.00-8.25)	2.31 (0.00-14.03)	0.93
Cornell VDP (mmms)	1548.5 ± 389.6	1661.5 ± 893.1	0.38
Sokolow (mm)	20.26 ± 5.91	19.72 ± 7.04	0.68
Ankle/brachial index	1.14 ± 0.11	1.14 ± 0.10	0.95
Carotid IMT average mean (mm)	0.74 ± 0.10	0.84 ± 0.13	<0.01
cf-PWV (m/sec)	8.63 ± 2.63	10.86 ± 2.05	<0.01
ba-PWV (m/sec)	13.18 ± 1.41	16.96 ± 3.44	<0.01
Central Augmentation Index	25.2 ± 14.8	30.0 ± 10.1	0.06
Peripheral augmentation index	90 (75–93)	92.5 (83–105)	0.04
Arteriole-venule ratio	0.73 ± 0.11	0.76 ± 0.12	0.29

CAVI: Cardio-Ankle Vascular Index. LDL: Low Density Lipoprotein. HDL: High Density Lipoprotein. HbA1C: Glycosylated Hemoglobin. SBP: Systolic Blood Pressure. DBP: Diastolic Blood Pressure. CVR: CardioVascular Risk. GFR: Glomerular filtration rate. MDRD-IDMS: Modification of Diet in Renal Disease-Isotopic Dilution Mass Spectrometry. VDP: Voltage–Duration Product. IMT: Intima-Media Thickness. cf-PWV: carotid femoral Pulse Wave Velocity. ba-PWV: brachial ankle Pulse Wave Velocity.

**Figure 1:**
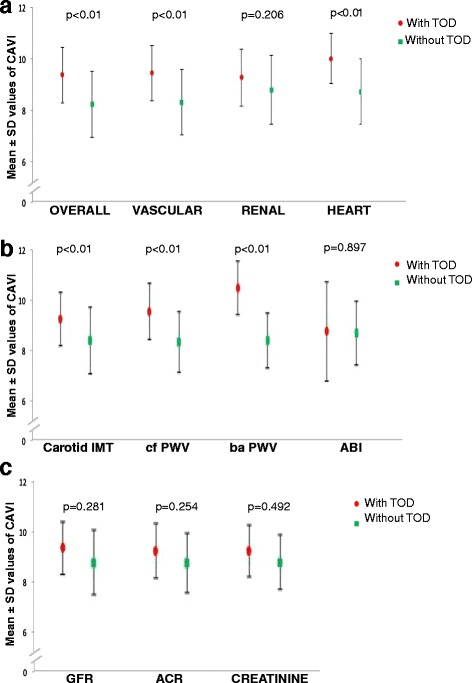
**Mean ± SD values of CAVI in patients with and without target organ damage. a**: Represents the mean values of CAVI ± SD between patients with any TOD and those without TOD (CAVI = 9.22 ± 1.1; vs. CAVI = 8.07 ± 1.3, p < 0.01); with vascular TOD and without it (CAVI = 9.29 ± 1.1; vs. CAVI = 8.15 ± 1.3, p = p < 0.01); with cardiac TOD and without it (CAVI = 9.86 ± 1.0; vs. CAVI = 8.56 ± 1.3, p = 0.002); and with renal TOD and without it (CAVI = 9.11 ± 1.1; vs. CAVI = 8.63 ± 1.3, p = 0.206). **b**: Represents the mean values of CAVI ± SD between patients with any TOD and those without TOD with different parameters: intima-media thickness (CAVI = 9.28 ± 1.08; vs. CAVI = 8.40 ± 1.33, p < 0.01) ; carotid femoral pulse wave velocity (CAVI = 9.58 ± 1.12; vs. CAVI = 8.35 ± 1.22, p < 0.01); brachial ankle pulse wave velocity (CAVI = 10.53 ± 1.08; vs. CAVI = 8.41 ± 1.11, p < 0.01) and Ankle-brachial index (CAVI = 8.77 ± 1.99; vs. CAVI = 8.69 ± 1.29, (p > 0.05). **c**: Represents the mean values of CAVI ± SD between patients with renal TOD and those without TOD with different parameters: glomerular filtration rate (CAVI = 9.26 ± 0.77; vs. CAVI = 8.66 ± 1.33, p > 0.05); creatinine (CAVI = 9.15 ± 1.24; vs. CAVI = 8.65 ± 1.32, p > 0.05); albumin creatinine ratio (CAVI = 9.14 ± 0.93; vs. CAVI = 8.68 ± 1.33, p > 0.05).

The difference in CAVI between patients with any TOD and patients without TOD was 1.15 (CI 95% 0.70 to 1.61, p < 0.001). This difference was 1.14 (CI95% 0.68 to 1.60, p < 0.001) in vascular TOD, 1.30 (CI 95% 0.51 to 2.10, p = 0.002) in the cardiac TOD, and 0.48 (CI 95% -0.27 to 1.22, p = 0.206) in renal TOD (Figure 1).

The CAVI has a positive correlation with age (r = 0.647; p < 0.01). The CAVI values had positive correlations with HbA1c (r = 0.30 p < 0.05) and systolic and diastolic blood pressure (r = 0.30, and r = 0.26 p < 0.05), which was adjusted for age, sex, and the presence of antihypertensive, lipid-lowering, and antidiabetic drugs. It had a negative correlation with waist circumference and body mass index (r = −0.13 and r = −0.24; p < 0.05).

In the multiple regression analysis, after adjustment for age, gender, cardiovascular risk, body mass index, and antihypertensive, antidiabetic, and lipid-lowering drugs, the CAVI as the independent variable showed a positive association with IMT (β = 0.29), cf-PWV (β = 0.83), ba-PWV (β = 2.12), CAIx (β = 3.42), and PAIx (β = 5.05) (p < 0.05, all comparisons). We have found no correlation between CAVI and ABI or AVR (Table 4).

**Table 4:** **Multiple regression analysis with TOD and vascular structure and function parameters as dependent variables and CAVI as independent variable**

**Dependent variable:**	**β**	**CI 95%**	**p value**
**IMT average mean**	0.29	0.09 to 0.48	<0.01
**ABI**	0.02	−0.002 to 0.04	0.08
**cf-PWV**	0.83	0.46 to 1.19	<0.01
**ba-PWV**	2.12	1.76 to 2.49	<0.01
**CAIx**	3.42	1.12 to 5.74	<0.01
**PAIx**	5.05	0.19 to 9.91	0.04
**AVR**	−0.01	−0.03 to 0.01	0.38

**Dependent variable:** IMT: Intima-Media Thickness of common carotid artery. ABI: Ankle Brachial Index. cf-PWV: carotid femoral Pulse Wave Velocity. ba-PWV: brachial ankle Pulse Wave Velocity. CAIx: Central Augmentation Index. PAIx: Peripheral Augmentation Index. AVR: ArterioVenous Ratio.

**Indepedent variable:** CAVI: Cardio-Ankle Vascular Index.

**Adjusted by:** Framingham D’Agostino cardiovascular risk score. Body mass index. Antihypertensive drugs. Lipid lowering drugs and antidiabetic drugs.

## Discussion

The results of this study show that the CAVI is positively associated with IMT, cf-PWV, ba-PWV, CAIx, and PAIx, regardless of cardiovascular risk and the drug treatment used. Patients with cardiovascular TOD have higher values of CAVI. Likewise, the CAVI was positively correlated with age, HbA1c, SBP, and DBP, and it was negatively correlated with waist circumference and body mass index.

Similar to the data found in this work, the CAVI was positively related with carotid IMT, cf-PWV, and ba-PWV in type 2 diabetes mellitus patients [37]. These results suggest that CAVI is a useful clinical marker for evaluating atherosclerosis in subjects with increased insulin resistance. Likewise, Kadota et al. [5] suggested the use of CAVI as a screening tool for atherosclerosis based on their findings from a general population study of 1014 adults showing strongly significant associations of CAVI scores with carotid intima-media thickness. Takaki et al. [25] compared the utility of these two parameters to detect arterial stiffness. Both CAVI and ba-PWV were significantly correlated with age and IMT. However, only CAVI was correlated with the parameters of left ventricular diastolic indices from echocardiography. Finally, only CAVI was significantly higher in the group with angina pectoris, and all parameters associated with atherosclerosis suggested that CAVI is superior to ba-PWV as a parameter of arterial stiffness.

Similarly we found an association between CAVI and ba-PWV or cf-PWV. The extent of atherosclerosis has been estimated using ba-PWV [6], ba-PWV are independently associated with the presence of coronary artery calcium (CAC) [44], a marker of preclinical atherosclerosis [45], but this can be influenced by blood pressure, and it is not very reproducible. Because the CAVI is independent of BP, highly reproducible, easy to apply, and does not require special techniques, its potential as a novel parameter of atherosclerosis has recently become recognized [12]. Izuara et al. [38] suggested that CAVI reflects systemic arterial sclerosis, including carotid atherosclerosis and coronary atherosclerosis, and that CAVI might be more useful for discriminating the probability of coronary atherosclerosis than findings of carotid atherosclerosis by high-resolution ba-PWV [29,46].

As far as we know, this is the first study that describes a positive association between the CAVI, the CAIx, and PAIx. Assessment of CAIx is a simple approach to quantify the role of wave reflection in determining an elevation of central blood pressure values [31,39]. Contrary to data published by Masugata et al. [47], who found a relationship between CAVI and the presence of left ventricule hypertrophy, this study found no relationship with either the left CAVI ventricule hypertrophy, renal TOD, the thickness of arteries and veins of the retina, or the ratio between the two.

In previous studies in Japanese population, has been described a negative association of CAVI with estimated glomerular filtration [48] and a positive correlation with the albumin creatinine ratio [49,50]. In our study, probably due to the low statistic power by the small sample size, we found no correlation with any of these parameters.

Consistent with previous studies, we found a positive correlation of CAVI with age, SBP, and DBP. Our study revealed that CAVI is highly correlated with age (r = 0.65), similar to the results reported by other authors for diabetic subjects [51,52] and for hypertensive diabetics (r = 0.63) [24]. A study that examined 32627 healthy residents from Japan showed that CAVI increases almost linearly with age from 20 to 70 years in males and females by 0.5 over 10 years [20].

The positive correlation between CAVI with SBP and DBP remains after adjusting for age, sex, and drug therapies used by patients. The results are consistent with those reported in diabetic patients [24]. However, in hypertensive patients, a correlation has only been found between CAVI and SBP, but not with DBP [20,28]. Some authors such as Nakamura et al. found no association of CAVI with blood pressure in patients with coronary disease [46]. These discrepancies suggest that the relationship of CAVI with the different components of blood pressure could be conditioned by previous disease presenting in the subjects analyzed.

Consistent with published results for patients with and without diabetes [52,53], the CAVI was positively correlated with HbA1c (r = 0.298, p < 0.05). In summary, these results suggest that CAVI is a good tool to detect the presence of vascular TOD, carotid atherosclerosis, and arterial stiffness in Caucasian patients with increased insulin resistance, and they may be helpful in clinical practice for this patient group, completing the results published by Takata et al. in 2013 for an Asian population [54].

### Limitations

The main limitation of this study was the source of the data for the cross-sectional study, which prevented us from establishing a temporal relationship between the CAVI and the different FRCV, TOD, and parameters that assess vascular function and structure during one week. Also, at the time of viewing these results, the subjects included in the study had multiple associated pathologies and were being treated with many drugs, which may have affected the CAVI values. We have tried to control this limitation by including the drugs most frequently used in the multiple regression analysis and in the correlation analysis as adjustment variables. Finally, the sample size of the individuals analyzed is not large.

## Conclusions

The results of this study suggest that the CAVI is positively associated with IMT, cf-PWV, ba-PWV, CAIx, and PAIx, regardless of cardiovascular risk and the drug treatment used. Patients with cardiovascular TOD have higher values of CAVI. This relationship between CAIx, PAIx, and CAVI opens new lines of research, since they measure different aspects of arterial stiffness and could improve the treatment of cardiovascular diseases.

## Abbreviations

ABI: Ankle-brachial index
AV: Arteriolar-venular
AVR: Arteriolar-venular ratio
ba-PWV: Brachial ankle pulse wave velocity
BMI: Body mass index
BP: Blood pressure
CAVI: Cardio ankle vascular index
CAIx: Central augmentation index
cf-PWV: Carotid femoral pulse wave velocity
Cornell VDP: Cornell voltage-duration product
DBP: Diastolic blood pressure
MDRD-IDMS: Modification of diet in renal disease-isotopic dilution mass spectrometry
GFR: Glomerular filtration rate
ICC: Intra-class correlation coefficient
IMT: Intima-media thickness
LVH: Left ventricular hypertrophy
PAIx: Peripheral augmentation index
PWV: Pulse wave velocity
TOD: Target organ damage
SBP: Systolic blood pressure
T2DM: Type 2 diabetes mellitus
